# mockrobiota: a Public Resource for Microbiome Bioinformatics Benchmarking

**DOI:** 10.1128/mSystems.00062-16

**Published:** 2016-10-18

**Authors:** Nicholas A. Bokulich, Jai Ram Rideout, William G. Mercurio, Arron Shiffer, Benjamin Wolfe, Corinne F. Maurice, Rachel J. Dutton, Peter J. Turnbaugh, Rob Knight, J. Gregory Caporaso

**Affiliations:** a Center for Microbial Genetics and Genomics, Northern Arizona University, Flagstaff, Arizona, USA; b Department of Biology, Tufts University, Medford, Massachusetts, USA; c Department of Microbiology & Immunology Department, Microbiome and Disease Tolerance Centre, McGill University, Montreal, Quebec, Canada; d Division of Biological Sciences, University of California San Diego, La Jolla, California, USA; e Department of Microbiology and Immunology, GW Hooper Foundation, University of California, San Francisco, San Francisco, California, USA; f Department of Computer Science and Engineering, University of California San Diego, La Jolla, California, USA; g Department of Pediatrics, University of California San Diego, La Jolla, California, USA; h Center for Microbiome Innovation, University of California San Diego, La Jolla, California, USA; i Department of Biological Sciences, Northern Arizona University, Flagstaff, Arizona, USA; University of Waterloo

## Abstract

Mock communities are an important tool for validating, optimizing, and comparing bioinformatics methods for microbial community analysis. We present mockrobiota, a public resource for sharing, validating, and documenting mock community data resources, available at http://caporaso-lab.github.io/mockrobiota/. The materials contained in mockrobiota include data set and sample metadata, expected composition data (taxonomy or gene annotations or reference sequences for mock community members), and links to raw data (e.g., raw sequence data) for each mock community data set. mockrobiota does not supply physical sample materials directly, but the data set metadata included for each mock community indicate whether physical sample materials are available. At the time of this writing, mockrobiota contains 11 mock community data sets with known species compositions, including bacterial, archaeal, and eukaryotic mock communities, analyzed by high-throughput marker gene sequencing.

**IMPORTANCE** The availability of standard and public mock community data will facilitate ongoing method optimizations, comparisons across studies that share source data, and greater transparency and access and eliminate redundancy. These are also valuable resources for bioinformatics teaching and training. This dynamic resource is intended to expand and evolve to meet the changing needs of the omics community.

## Abstract (precis)

The availability of standard and public mock community data will facilitate ongoing method optimizations, comparisons across studies that share source data, and greater transparency and access and eliminate redundancy. These are also valuable resources for bioinformatics teaching and training. This dynamic resource is intended to expand and evolve to meet the changing needs of the omics community.

## INTRODUCTION

Important steps in the development of bioinformatics methods are the identification and acquisition of useful test data sets. For microbiome bioinformatics tools, test data sets frequently take the form of simulated data (1–3), data from natural microbial communities that are considered to be well understood (3), or “mock community” (MC) data (4–6). Each of these data types has its own pros and cons. With simulated data, a model is developed to generate artificial data computationally, e.g., simulated marker gene sequence reads. Because the developer of the simulated data has complete control over the model, the true values of the optimization criteria, e.g., the relative abundance of different species in a sample, are known with certainty. Although this is very useful, optimizing a method on simulated data fits the method to work well on the results of the model used for simulation, and hence, simulated data must incorporate technologically relevant error models (1, 2). This can be problematic if the model is not a good representation of reality, e.g., if appropriate error models have not yet been developed for a new technology/version. Natural microbial communities are on the opposite end of the spectrum. Assumptions are not made in generating the data but are made about the true values of optimization criteria because the right answer, the actual composition of a natural community, is not necessarily known. Mock microbial communities attempt to provide a balance between these two types of test data for microbiome method benchmarking by providing data on samples of actual biological origin but also of a known composition. Thus, MC sample data are technologically relevant (i.e., represent actual experimental observations) to enable method evaluations under actual working conditions. It is important to stress that none of these approaches is perfect, and combining multiple test data types is common and likely to provide insight beyond evaluations using either data type on its own.

An MC is a defined mixture of known microbial strains. To make an MC, axenic cultures are deliberately combined in precise ratios such that the species composition is known. If the genomes of these strains are sequenced, the expected collective gene content can be inferred, yielding a mock metagenome (7). This mixture is then processed as if it were a natural community sample, including DNA extraction, amplification of marker genes such as 16S rRNA genes (if applicable), and sequencing. This allows the generation of real sequence data, nullifying concerns about assumptions made during the generation of simulated data sets, and provides known optimization criteria, though in practice, some uncertainty is still present. One limitation of mock communities, however, is that they often are composed of few taxa relative to natural microbial communities, so overfitting of methods to unrealistic conditions is still possible, emphasizing the importance of employing different types of test data, as was done previously (3, 6). MCs have been widely used in microbiome method development, including development of sequencing protocols (4, 8, 9), validation of sequence quality control (5, 10–12), and evaluation and comparison of bioinformatics methods for marker gene (13, 14) and metagenomics sequencing (7, 15).

We consider MCs to be composed of three parts. First, the physical sample materials consist of microbial cells, DNA, RNA, or other biological materials, which are not hosted by mockrobiota. Second, expected composition data comprise taxonomy or gene annotations and abundances and reference sequences of community members, e.g., 16S rRNA gene sequences. The third component is raw data, such as raw sequence data obtained from marker gene sequencing of the MC. MCs are a valuable community resource, and public sharing of standardized MC data will facilitate ongoing method improvements for the omics community, direct comparisons among studies that share source MC data, and greater transparency and access to source MC data and eliminate redundancy, as developers can bypass the time-consuming task of generating new MCs if appropriate MC data sets already exist. The use of multiple test data sets is advisable to generalize method optimization across different conditions (16) and to avoid overfitting, underlining the value of standard and public MCs to accelerate bioinformatics method development.

## RESULTS AND DISCUSSION

We present mockrobiota, a public resource for sharing, validating, and documenting MC resources. mockrobiota is open source and hosted on GitHub, an online software revision control and collaboration tool. The materials contained in mockrobiota include data set and sample metadata, expected composition data that are annotated on the basis of one or more reference taxonomies, links to raw data for each MC data set, and reference sequences for MC members. Reference sequences are optional but are strongly encouraged, as these greatly enhance the usefulness of the associated MC, as discussed below. mockrobiota does not supply physical sample materials directly, but the data set metadata included for each MC indicate whether physical sample materials are available from the contributor. If so, relevant contact information is listed for requesting that material directly from the contributor. Because of storage limits, raw sequence data are stored not in mockrobiota itself but rather in other public resources such as FTP servers or the QIITA database (https://qiita.ucsd.edu/) and linked directly from the GitHub repository. These links to raw data are automatically validated regularly as described below.

**FIG 1:**
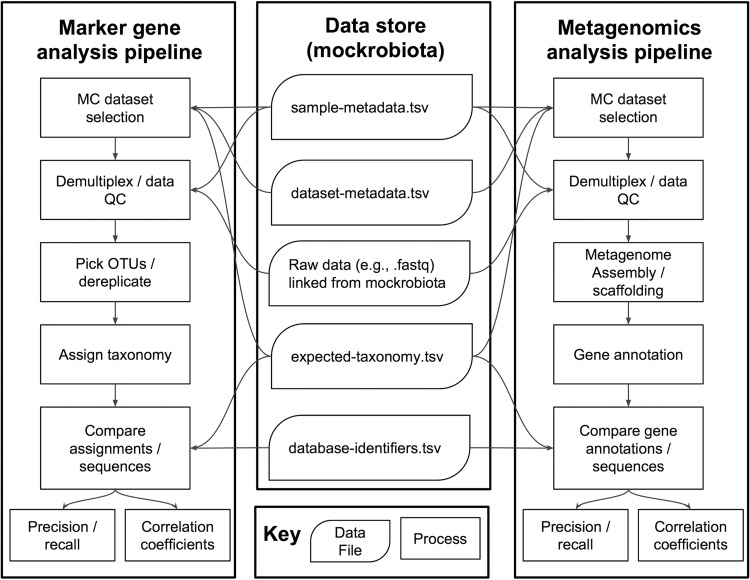
Example usage of mockrobiota MC resource for marker gene and metagenome sequencing pipelines. MC data sets are selected on the basis of multiple input criteria, including data set metadata, sample metadata, and represented taxa. Raw data (e.g., fastq) are demultiplexed, sequences are dereplicated or clustered as operational taxonomic units (OTUs) (marker gene data) or assembled/scaffolded to template genomes (metagenome data), and representative sequences are annotated (e.g., by taxonomy or gene). Observed taxonomic/gene annotations and abundances are compared to the expected composition (expected taxonomic assignments/gene annotations and abundances) of that MC, e.g., to generate precision and recall scores or correlations between observed and expected values. QC, quality control.

At the time of this writing, mockrobiota contains 11 MCs with known species compositions (including bacterial, archaeal, and eukaryotic MCs) analyzed by high-throughput marker gene sequencing (Table 1). Known taxonomies of these samples are annotated with Greengenes (17) and SILVA (18) reference taxonomies for bacterial/archaeal samples, SILVA for eukaryote samples, and UNITE (19) for fungus-only eukaryote samples. Translating from an MC developer’s taxonomic description of a sample to relevant taxonomic database annotations can be time-consuming and error prone. The availability of these annotations in mockrobiota will therefore save time and increase consistency across studies that use these data. MCs can be utilized in a few simple steps (Fig. 1).

**TABLE 1:** Marker gene sequencing MCs currently available in mockrobiota

Data set	Target region^a^	Read length (nucleotides)	Method	Sample count(s)^b^	Strain count	Original citation
Mock-1	16S	100	HiSeq	1 E	48	5
Mock-2	16S	150	MiSeq	1 E	48	5
Mock-3	16S	250	MiSeq	2 E, 2 S	22	5
Mock-4	16S	150	MiSeq	2 E, 2 S	22	5
Mock-5	16S	250	MiSeq	2 E, 2 S	22	14
Mock-6	16S	100	GAIIx	3 E	67	6
Mock-7	16S	100	HiSeq	3 E	67	21
Mock-8	16S	100	HiSeq	3 E	67	14
Mock-9	ITS	100	HiSeq	3 E	16	14
Mock-10	ITS	100	HiSeq	3 E	16	14
Mock-11	18S	90	HiSeq	1 E	12	5

^a^ Marker gene sequence target. 16S, 16S rRNA gene; ITS, internal transcribed spacer; 18S, 18S rRNA gene.

^b^ Number of MC samples contained in MC data set. E, samples with even abundance ratios among strains; S, samples with staggered (uneven) abundance ratios.

Attributes of each MC are summarized in data set metadata tables viewable in the mockrobiota repository and linked from a master table (https://github.com/caporaso-lab/mockrobiota/blob/master/inventory.tsv), facilitating navigation and selection of the MCs that best fit users’ needs. From these tables, users can directly access links for downloading MC data, metadata, and auxiliary files. The repository also contains guidelines for formatting and contributing new resources to mockrobiota (https://github.com/caporaso-lab/mockrobiota/blob/master/CONTRIBUTING.md). All core MC resources are available in common file formats to facilitate universal access without any specific software requirements. This allows end users to “plug and play” their MCs of choice into analysis pipelines without software bottlenecks, though it does not guarantee compatibility with all analysis software/versions. For example, mockrobiota does not update raw sequence data to conform to changing formatting standards, because raw data files are hosted externally.

mockrobiota currently requires expected observation data in the form of sequence annotations, e.g., taxonomy or gene annotations, but we strongly recommend additionally submitting reference sequences in the form of accession numbers. Reference sequences are more useful expected observations for many applications, e.g., quality control of sequencing data (12); they forgo the need for annotations, which are static and database specific, and they eliminate the risk of inaccurate annotations, e.g., following taxonomy changes or if original source strains were incorrectly identified. However, not all applications of MCs rely on reference sequences and reference sequences are not available for all of the source strains in all of the mock communities currently hosted on mockrobiota. Hence, reference sequence accession numbers remain an optional data category.

Importantly, mockrobiota makes use of Travis CI (https://travis-ci.org/) for continuous integration testing to ensure data integrity. Any time a change to any of the mockrobiota files is proposed, e.g., modification of an existing data set or the addition of a new MC, a series of tests is run to validate all of the data. This includes confirming that raw data links are valid and accessible, that files hosted in the mockrobiota GitHub repository are formatted correctly, and that expected taxonomic relative abundances in each sample sum to 1.000. Together, these ensure that users can always access the data in mockrobiota (i.e., links are not outdated) and that all MC data are reliable and available in consistent formats, facilitating analyses that involve multiple MCs. This model of using software testing approaches to validate community data resources would be very useful to generally adopt in bioinformatics and, as illustrated here, is now simple to implement with free continuous-integration testing systems.

Hosting mockrobiota on GitHub provides an additional major benefit in that the data are not static. This resource will grow and evolve to meet the needs of the omics community as more MCs are contributed and to conform to changes in related resources, such as reference sequence databases and taxonomic annotations. Finally, hosting on GitHub invites community involvement to contribute, update, revise, and evaluate MC resources.

MCs provide many benefits for bioinformatics method benchmarking, complementing the use of other test data (e.g., natural and simulated communities). We anticipate that a public MC database will eliminate redundant effort and improve consistency across studies that use the same MCs and thereby facilitate method advances for the benefit of the entire microbiome research community. Standard MCs are also useful for teaching and training, providing reliable test cases with expected observations against which students and researchers can hone their skills. We hope that mockrobiota will fill these gaps and that community members will contribute to the growth and development of this resource.

## MATERIALS AND METHODS

### Data availability.

Links to raw data, database and sample metadata, expected composition data, and other useful resources are hosted in a public GitHub repository, which can be found at https://github.com/caporaso-lab/mockrobiota.

mockrobiota is a data resource and does not provide physical samples (e.g., DNA, RNA, cell mixtures) of MCs. However, contributors are encouraged to share physical samples of their mock communities as supplies permit. The data set metadata included for each MC indicate whether physical sample materials are available to be shared and, if so, list relevant contact information for requesting that material directly from the contributor.

### Expected observation data generation.

Expected observation data, representing the known composition of an MC, are provided in mockrobiota in two forms, source data and expected composition (taxonomy or gene annotation) data. Source data provide a record of the original inputs to the MC as a list of microbial strains and their relative abundances. Ideally, a strain ID should be provided to identify a retrievable source strain, allowing accurate tracking and revision of taxonomic information. These data are generally created by the developer of the MC, and taxonomic groups are not necessarily annotated with respect to any specific taxonomic reference database. An example of source data is given in Table 2, and a template example is provided in the mockrobiota data directory. These files consist of two or more columns. The first column (Taxonomy) lists the taxonomy of each MC member in as much detail as can be provided by the MC developer. In Table 2, this contains the genus, species, and strain ID of each strain added to the MC on separate lines. The remaining columns each represent an individual MC sample contained within the data set. The column heads contain the names of the samples and must correspond to the sample names listed in the sample-metadata.tsv file for that data set. The values in the column are the relative abundances at which each taxon is present in the samples.

**TABLE 2:** Example source composition

Taxonomy	Sample 1
*Staphylococcus aureus* ATCC BAA-1718	0.200
*Staphylococcus epidermidis* ATCC 12228	0.200
*Streptococcus agalactiae* ATCC BAA-611	0.200
*Streptococcus mutans* ATCC 700610	0.200
*Streptococcus pneumoniae* ATCC BAA-334	0.200

Expected composition data represent the known composition of the MC (e.g., taxonomies or KEGG pathways) annotated according to a specific reference database. Like source data files, expected composition data files are created and carefully reviewed by contributors to mockrobiota; the automatic integrity checks employed by mockrobiota cannot ensure that expected observation annotations are accurate. It is in the interest of contributors to ensure the accuracy of their data sets, as poor curation will deteriorate the quality of results obtained when using a given MC, decreasing the likelihood that the MC will be used and cited by other researchers. Compilation of expected composition data is not a trivial task and requires careful review of database annotations to ensure that accurate annotations are applied to source data. An example of expected composition data is shown in Table 3, corresponding to the source data example shown in Table 2. In these files, column layout and header naming follow the same conventions as described above for source files. The first column (Taxonomy) lists taxonomic descriptions or other annotations associated with each species added to the MC. These taxonomic descriptions (or other annotations) are drawn from an appropriate reference database, e.g., the Greengenes (17) or SILVA (18) rRNA gene sequence database. The taxonomic description should be copied directly from the reference database. If using this MC for comparison of expected versus observed taxonomy assignments, the same reference database must be used for taxonomy assignment of the MC sequences during analysis to allow for direct comparison between expected and observed results. The expected composition data are deposited in mockrobiota in a directory structure that indicates the reference database name and version used for annotation. For example, expected composition data that list taxonomy strings from the Greengenes 13_8 release (17) are deposited in mockrobiota/data/mock-X/greengenes/13_8/expected-taxonomy.tsv, where mock-X is the number assigned to that MC.

**TABLE 3:** Example expected composition, annotated with Greengenes 13_8 reference taxonomy

Taxonomy	Sample 1
k__Bacteria;p__Firmicutes;c__Bacilli;o__Bacillales;f__Staphylococcaceae;g__Staphylococcus;s__aureus	0.200
k__Bacteria;p__Firmicutes;c__Bacilli;o__Bacillales;f__Staphylococcaceae;g__Staphylococcus;s__epidermidis	0.200
k__Bacteria;p__Firmicutes;c__Bacilli;o__Lactobacillales;f__Streptococcaceae;g__Streptococcus;s__	0.400
k__Bacteria;p__Firmicutes;c__Bacilli;o__Lactobacillales;f__Streptococcaceae;g__Streptococcus;s__agalactiae	0.200

Several issues may arise during database annotation that require careful attention, and hence, careful manual curation of expected composition files is important. Specific taxa may not be represented in a reference taxonomy at the species level and must be annotated to the nearest common lineage. For example, *Streptococcus mutans* and *Streptococcus pneumoniae* in the source data (Table 2) are annotated as g__Streptococcus;s__ in the expected composition example above (Table 3).

Multiple input strains, listed as separate entities in the source files, may need to be combined under common annotations in the expected composition files if they are not listed in the reference database. The relative abundance of an expected taxonomy will be equal to the sum of all of the members matching that taxonomy. For example, multiple strains may be combined as a single species, or species not listed in the reference database may be combined under a single genus; note the relative abundance of g__Streptococcus;s__ listed in the example above.

Reference databases may contain quirks that complicate the annotation of expected composition files, such as listing strain IDs or different taxonomic lineages for multiple entries of the same species. MC developers should carefully inspect reference database annotations and all expected composition files. The accuracy of taxonomic descriptions cannot be checked (i) by mockrobiota’s automatic integrity checks, because all possible databases that could be used for annotation will not be available to the testing system, or (ii) by mockrobiota’s developers during pull request reviews. Ultimately, the integrity of each data set is the responsibility of the contributor.

Expected composition data will consist of one of two types. The first is a marker gene MC (expected taxonomic composition of a mixture of microbial cells). The taxonomic annotations present in the expected data will be specific to the database version that is used for analysis and will be meaningless if used for different database versions. Likewise, they may not match the source annotation, i.e., the taxonomy of each strain to the best knowledge of the MC’s creator, if taxonomic annotations have been revised or if the reference database being used does not contain a given taxonomy. The second is a metagenome MC (expected gene composition of a mixture of microbial cells/genomes). Gene annotations will be reference database specific, as for the marker gene MCs described above.

Other MC data types are theoretically possible and could be included in mockrobiota, which only defines required information, files, and file formats. Expected data definitions can expand as other MC data types are contributed to mockrobiota.

The MCs currently deposited in mockrobiota are all marker gene MCs representing known compositions of microbial species analyzed by marker gene sequencing methods. Taxonomy strings for 16S rRNA gene MCs (mock-1 through mock-8) were generated with the Greengenes 13_8 release (17) and the SILVA 119 release (18) sequence reference databases, both prefiltered to 97% sequence identity. Taxonomy strings for fungal internal transcribed spacer MCs (mock-9 and mock-10) were generated with the UNITE+INSD database (9/24/12 release) (19) prefiltered at 97% identity and from which sequences with incomplete taxonomy strings and empty taxonomy annotations (e.g., “uncultured fungus”) were removed as described previously (20). Taxonomy strings for the 18S rRNA MC (mock-11) were generated with the SILVA 119 release (18).

### Data set metadata.

Data set metadata are provided in the base directory for each MC data set in the dataset-metadata.tsv file. These metadata include important features about the generation of the MC, citation information, and data accessibility information. The required fields and their definitions are listed at https://github.com/caporaso-lab/mockrobiota/blob/master/CONTRIBUTING.md.

### Sample metadata.

Sample metadata files list the metadata associated with individual samples. The example sample metadata file (https://github.com/caporaso-lab/mockrobiota/blob/master/data/example-1/sample-metadata.tsv) includes all of the required fields. The first is SampleID; the sample ID should be a unique identifier for each sample. The second is BarcodeSequence, which is the unique barcode/index sequence associated with that sample. This will be needed to demultiplex relevant data from the raw data files. Only IUPAC DNA characters are acceptable. The third is LinkerPrimerSequence, which is the full forward PCR primer sequence (including any “linker” sequences) used to amplify gene targets from that sample (if applicable). Only IUPAC DNA characters are acceptable. For nonmarker gene studies, list “NA.” The fourth is ReversePrimer, which is the reverse PCR primer sequence used to amplify gene targets from that sample (if applicable). Only IUPAC DNA characters are acceptable. For nonmarker gene studies, list “NA.” The fifth is PrimerName, the common name of the primer pair used to amplify gene targets from that sample (if applicable) in the format (forward primer)f-(reverse primer)r, for example, 515f-806r. For nonmarker gene studies, list “NA.” The sixth is Description, a short, unique description of the sample. As the README.md and dataset-metadata.tsv files contained within each MC data set list longer descriptions of the sample(s) included in that data set, this field should be used primarily to indicate distinguishing features associated with each sample.

### Raw data generation.

Raw data for MCs fall into different types, corresponding to the MC types and expected composition data defined above, i.e., Marker gene MC (raw data consisting of marker gene sequences) and Metagenome MC (raw data consisting of shotgun metagenome sequences).

All raw sequence data are currently linked in fastq format. mockrobiota does not host raw data files and only ensures that valid, accessible links are provided in the data set metadata. MC data sets that contain multiple samples are provided in nondemultiplexed files, i.e., one file per sequencing run, containing multiple uniquely barcoded samples. All raw data files are archived by using the standard gzip compression format, and index/barcode sequences are provided as a separate fastq file. Reverse sequencing reads are accepted but not required. All submissions must conform to the standard file names mock-forward-read.fastq.gz, mock-reverse-read.fastq.gz, and mock-index-read.fastq.gz.

The raw data for each marker gene MC currently available in the repository were generated by 11 separate sequencing runs on the Illumina GAIIx (*n* = 1), HiSeq 2000 (*n* = 6), and MiSeq (*n* = 4), as described in Table 1 and in the dataset-metadata.tsv files associated with each data set in mockrobiota. These consisted of genomic DNA from known species isolates deliberately combined at defined rRNA copy number ratios.
